# Osteopontin Is a Novel Downstream Target of SOX9 With Diagnostic Implications for Progression of Liver Fibrosis in Humans

**DOI:** 10.1002/hep.25758

**Published:** 2012-09

**Authors:** James Pritchett, Emma Harvey, Varinder Athwal, Andrew Berry, Cliff Rowe, Fiona Oakley, Anna Moles, Derek A Mann, Nicoletta Bobola, Andrew D Sharrocks, Brian J Thomson, Abed M Zaitoun, William L Irving, Indra N Guha, Neil A Hanley, Karen Piper Hanley

**Affiliations:** 1 Endocrinology and Diabetes Group, School of Biomedicine, University of ManchesterManchester, United Kingdom; 2 Liver Research Group, Institute of Cellular Medicine, University of NewcastleNewcastle-upon-Tyne, United Kingdom; 3 School of Dentistry, University of ManchesterManchester, United Kingdom; 4 Faculty of Life Sciences, University of ManchesterManchester, United Kingdom; 5 School of Molecular Medical Sciences, Nottingham Digestive Diseases Center, Biomedical Research Unit in Gastroenterology and Liver Disease, University of Nottingham and Nottingham University Hospitals, Queens Medical Center CampusNottingham United Kingdom; 6 Department of Cellular Pathology, Nottingham Digestive Diseases Center, Biomedical Research Unit in Gastroenterology and Liver Disease, University of Nottingham and Nottingham University Hospitals, Queens Medical Center CampusNottingham United Kingdom; 7 Liver Unit, National Institute of Health Research, Nottingham Digestive Diseases Center, Biomedical Research Unit in Gastroenterology and Liver Disease, University of Nottingham and Nottingham University Hospitals, Queens Medical Center CampusNottingham United Kingdom

## Abstract

Osteopontin (OPN) is an important component of the extracellular matrix (ECM), which promotes liver fibrosis and has been described as a biomarker for its severity. Previously, we have demonstrated that Sex-determining region Y-box 9 (SOX9) is ectopically expressed during activation of hepatic stellate cells (HSC) when it is responsible for the production of type 1 collagen, which causes scar formation in liver fibrosis. Here, we demonstrate that SOX9 regulates OPN. During normal development and in the mature liver, SOX9 and OPN are coexpressed in the biliary duct. In rodent and human models of fibrosis, both proteins were increased and colocalized to fibrotic regions *in vivo* and in culture-activated HSCs. SOX9 bound a conserved upstream region of the *OPN* gene, and abrogation of *Sox9* in HSCs significantly decreased OPN production. Hedgehog (Hh) signaling has previously been shown to regulate *OPN* expression directly by glioblastoma (GLI) 1. Our data indicate that in models of liver fibrosis, Hh signaling more likely acts through SOX9 to modulate *OPN*. In contrast to Gli2 and Gli3, Gli1 is sparse in HSCs and is not increased upon activation. Furthermore, reduction of GLI2, but not GLI3, decreased the expression of both SOX9 and OPN, whereas overexpressing SOX9 or constitutively active GLI2 could rescue the antagonistic effects of cyclopamine on OPN expression. *Conclusion*: These data reinforce SOX9, downstream of Hh signaling, as a core factor mediating the expression of ECM components involved in liver fibrosis. Understanding the role and regulation of SOX9 during liver fibrosis will provide insight into its potential modulation as an antifibrotic therapy or as a means of identifying potential ECM targets, similar to OPN, as biomarkers of fibrosis. (Hepatology 2012;56:1108–1116)

Fibrosis of the liver is characterized by excessive extracellular matrix (ECM) deposition. One of the major cell types responsible for this is the hepatic stellate cell (HSC).^1^, ^2^ In response to injury, HSCs become activated into proliferative myofibroblasts, migrate into the surrounding parenchymal cells, and secrete tissue-damaging ECM, the major component of which is type 1 collagen (COL1). In addition, the ECM contains a complex mix of proteins that promote cell proliferation, migration, and differentiation. One ECM component with such roles is the matricellular glycophosphoprotein, osteopontin (OPN), also known as secreted phosphoprotein 1.

OPN is detected in a wide range of tissues and body fluids, with physiological roles during development (e.g., in bone, bile duct formation, and during vascular remodeling), immune system regulation, and wound repair.^3^ However, it is also associated with pathological processes relating to cancer and inflammation.^3^, ^4^ The ability of OPN to mediate such diverse cellular functions is likely related to its extensive post-translational modifications and ability to signal through several integrin and CD44 variant receptors.^3^, ^5^

OPN contributes to wound scarring in skin^6^ and has been implicated in lung, kidney, and heart fibrosis.^7^-^9^ It has previously been detected in activated HSCs, where it is thought to mediate cell migration.^10^ More recently, OPN levels have been highlighted as a potential biomarker of liver disease, levels correlating with the severity of disease,^11^-^13^ and has been reported to promote the progression of fibrosis in nonalcoholic steatohepatitis.^14^ The latter study, and others,^15^ has demonstrated regulation of *OPN* expression by Hedgehog (Hh) signaling, mediated by the member of the glioblastoma (GLI) family of transcription factors, GLI1, binding to an upstream element of the *OPN* promoter.^15^ There are three GLI transcription factors, with different activator and repressor forms of GLI2 and GLI3 generated by alternative splicing of the parent transcripts.^16^

Previously, we have shown that the transcription factor, sex-determining region Y-box 9 (SOX9), becomes ectopically expressed in activated HSCs, where it is responsible for COL1 production.^17^ During development, SOX9 has diverse roles regulating the expression of a number of genes encoding ECM proteins.^18^ SOX9 has also been associated with fibrotic pathologies affecting the skin, kidney, and heart.^18^-^23^

In this present study, we show that OPN and SOX9 colocalize to biliary cells in the healthy liver and to the same regions of fibrotic tissue. Both are markedly increased during HSC activation, when it appears unlikely that GLI1 regulates *OPN*. Instead, we demonstrate that SOX9 lies downstream of GLI2 and is responsible for *OPN* expression. These data support a role for SOX9 during the progression of liver fibrosis as a regulator of key fibrotic ECM components, and suggest that the manipulation of SOX9 or its downstream targets may be a means of developing antifibrotic therapies. Furthermore, the identification of other ECM targets of SOX9 may have additional utility as biomarkers of fibrotic severity in liver disease similar to recent studies on OPN.^11^, ^12^

## Materials and Methods

### Human Tissue and Serum Collection

Human fetal material was collected under guidelines issued by the Polkinghorne Committee, as described previously.^17^, ^24^ Ethical approval was granted by the North West Regional Ethics Committee. Freshly isolated adult liver was purchased after resection (Invitrogen Ltd., Warrington, UK) and processed as previously described.^17^, ^24^

### Animal Models of Liver Fibrosis

Liver fibrosis was induced by 5-week treatment of adult male Sprague-Dawley rats with CCl_4_^25^ or in C57Bl/6 mice fed a methionine- and choline-deficient diet for 7 weeks.

### Immortalized and Primary Cell Culture

Primary rat hepatic stellate cells (rHSCs) were isolated as described previously.^17^, ^25^ Human LX2 cells were a gift from Prof. Scott Friedman (Mount Sinai School of Medicine, New York, NY).^26^ Primary human HSCs (hHSCs) were isolated after liver resection (see Supporting Materials and Methods) under ethical approval from the Nottingham Research Ethics Committee, activated in culture, and fixed for immunocytochemistry (ICC).^17^ All cells were cultured in monolayer at 5% CO_2_ and 37°C in Dulbecco's modified Eagle's medium plus L-glutamine, Na-pyruvate, and antibiotics supplemented with fetal bovine serum: 1% (low serum) or 10% (high serum) for LX2 cells, as indicated, or 16% for rHSCs and 10% for hHSCs.^17^ Gene silencing was carried out transiently using short interfering RNA (siRNA) (see Supporting Table 1) with HiPerFect (LX2 cells) or Nucleofection for HSCs (Amaxa Biosystems GmbH, Cologne, Germany), as described previously.^17^ To interrogate Hh signaling, LX2 cells and rHSCs were treated with 5 μM of 3-Keto-N-(aminoethyl-aminocaproyl-dihydrocinnamoyl)/cyclopamine (CYC) (Merck Chemicals Ltd., Nottingham, UK) or 100 and 50 nM of smoothened agonist (SAG; Merck Chemicals Ltd.) for LX2 cells and HSCs, respectively. SAG treatments were performed in serum-free conditions. Overexpression experiments were carried out in LX2 cells. Plasmid transient transfections were achieved using Transfast reagent (Promega, Madison, WI), as described previously,^17^ in the presence or absence of CYC (described above). Briefly, 0.5 μg of expression plasmids (see Supporting Table 2) containing SOX9 or myc-tagged constitutively active GLI2 (GLI2ΔN)^27^ or active GLI3 (GLI3A-myc)^28^, ^29^ were transiently transfected into LX2 cells. After 24 hours, cells were then treated with CYC or vehicle control for an additional 24 hours and assayed for protein expression. All experiments were carried out with the appropriate empty vector (EV) control.

### Gene Expression, Protein Analysis, and Chromatin Immunoprecipitation Assays

Antibodies used are listed in Supporting Table 3. Tissue preparation, immunohistochemistry (IHC), ICC, immunoblotting, and quantification were performed as described previously.^17^ For quantitative reverse-transcription polymerase chain reaction (qPCR), RNA was isolated from cells using the RNeasy kit (Qiagen Ltd., West Sussex, UK). Complementary DNA (cDNA) was synthesized from 1 μg of RNA with a RNA-to-cDNA kit (Applied Biosystems Ltd., Cheshire, UK). qPCR reactions were carried out on a StepOnePlus Real-Time PCR system (Applied Biosystems Ltd) using 1 μL of cDNA, intron-spanning primers, wherever possible (Supporting Table 4), and SYBR green (PrimerDesign Ltd., Southampton, UK). *GusB* was used as a housekeeper control for gene expression, as described previously.^30^ Changes in messenger RNA (mRNA) expression were calculated using ^ΔΔ^C_T_ methodology. Chromatin immunoprecipitation (ChIP) assays were performed as described previously.^31^, ^32^ Further details are described in the Supporting Materials and Methods.

### Statistical Analysis

Statistical significance was determined by the two-tailed Student *t* test. All experiments were carried out three times or more (n = 3). For rHSCs, experimental data arise from three different preparations of stellate cells from different animals. Error bars in graphs show the standard error for each experiment.

## Results

### Expression of SOX9 and OPN in Biliary Duct and Liver Fibrosis in Humans and Rodents

**Fig. 1:**
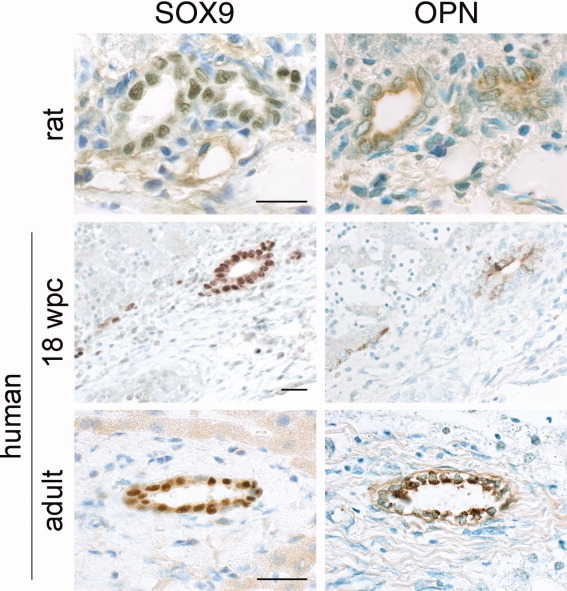
IHC of SOX9 and OPN in healthy liver. Consecutive 5-μm sections of healthy liver in rat and human (at 18 weeks post-conception [wpc] and adulthood) stained for SOX9 and OPN (brown) and counterstained with toludine blue. Note detection only in the round nuclei (SOX9) and cytoplasm (OPN) of biliary epithelial cells. Size bar represents 50 μm.

**Fig. 2:**
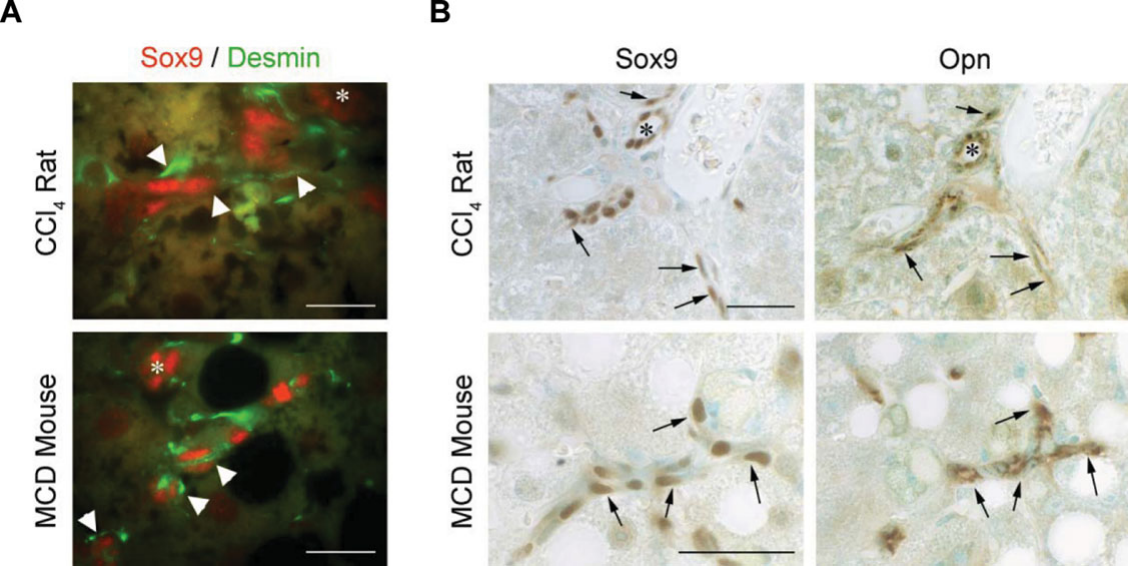
IHC of SOX9, OPN, and desmin in fibrotic liver. (A) Dual IF in fibrotic tissue from rat and mouse showing nuclear Sox9 (red) in biliary cells (asterisks) and in cells with cytoplasmic staining for desmin (green) (white arrowheads). (B) Consecutive 5-μm tissue sections shown from fibrotic rat and mouse liver stained with Sox9 and Opn (brown) counterstained with toluidine blue. Note similarly located staining for Sox9 and Opn in cells with more spindle-shaped nuclei (arrows) as well as in biliary cells (asterisk). Size bars represent 100 μm.

**Fig. 3:**
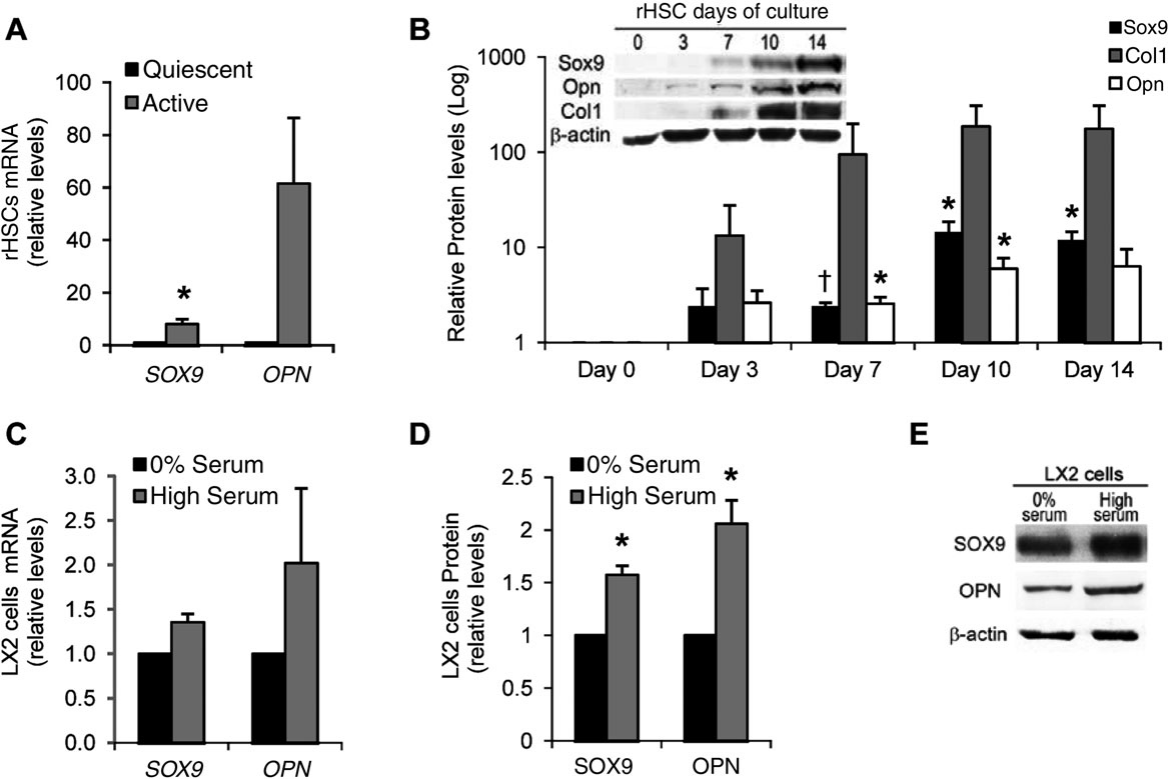
SOX9 and OPN expression in activated HSCs. (A-E) Quantification of SOX9 and OPN in quiescent and activated rHSCs and LX2 cells by qPCR (A and C) and immunoblotting (B, D, and E) (in [A], *Sox9* was up-regulated 8.0-fold). In (B), induction of Sox9, Opn, and Col1 is shown during activation of rHSCs in culture (relative to quiescent; day 0). Representative immunoblotting images for (B) and (D) are shown as inset (B) or as an individual image (E), respectively. All immunoblotting quantification was normalized to β-actin. *P < 0.05; †*P* < 0.005, compared to quiescent day 0 cells (A and B) or 0% serum (C and D).

**Fig. 4:**
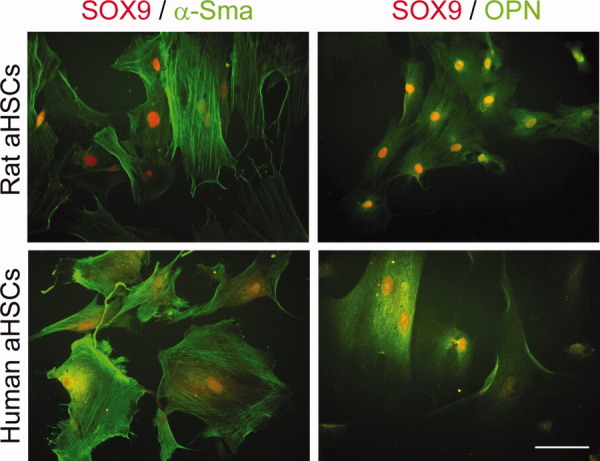
SOX9 and OPN expression in activated hHSCs and rHSCs. IF showing nuclear SOX9 (red) and cytoplasmic α-SMA (green; left panel) or OPN (green; right panel) in rat and human activated HSCs (aHSCs). Size bar represents 20 μm.

SOX9 was detected in the round nuclei of biliary epithelial cells in fetal and adult livers, where it demonstrated cellular colocalization with OPN (Fig. 1 and Supporting Fig. 1). Previous data have independently identified OPN^11^, ^12^, ^14^ and SOX9^17^ in areas of liver fibrosis in animal models. Here, in rat and mouse models of liver fibrosis, nuclear Sox9 localized to desmin-positive cells, confirming its presence in HSCs (Fig. 2A). Opn localized with Sox9 to spindle-shaped HSCs with elongated nuclei in areas of fibrosis as well as to biliary cells (Fig. 2B). *In vitro*, Opn was barely detected in quiescent rHSCs that lacked Sox9 (Fig. 3A,B and Supporting Fig. 2A,B). However, as reported by others,^10^, ^14^*Opn* expression was induced ∼60-fold and its protein increased as rHSCs became activated on tissue culture plastic over 2 weeks, paralleling the induction of Sox9 and the sequential increase in Col1 (Fig. 3A,B). Similar results were gained using the human cell line, LX2, an *in vitro* model of stellate cells.^26^ In high-serum conditions, which mimic stellate cell activation, OPN was increased along with SOX9 (Fig. 3C-E). Final confirmation of OPN cellular colocalization with SOX9 in both activated rHSCs and hHSCs was demonstrated *in vitro* using immunofluorescence (IF). Nuclear SOX9 is shown surrounded by OPN or α-smooth muscle actin (α-SMA) (Fig. 4). These data led us to question whether SOX9 was capable of regulating *OPN*.

### Sox9 Is Responsible for Opn Expression in Activated HSCs

**Fig. 5:**
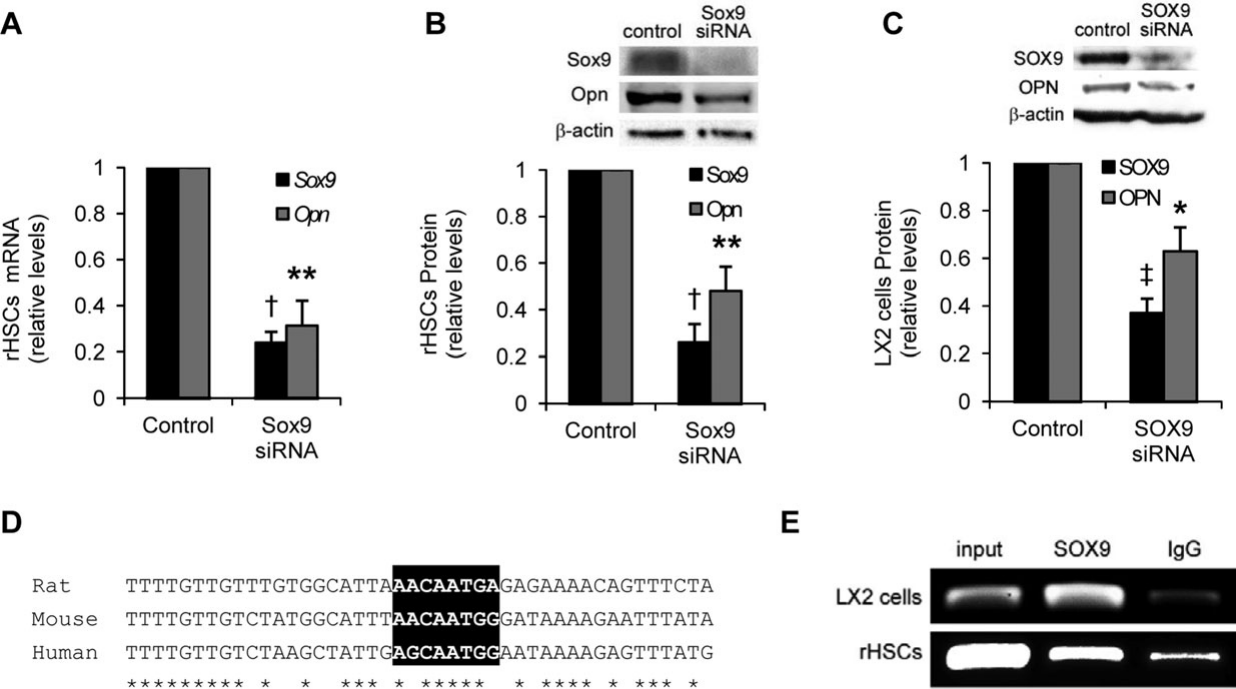
SOX9 regulation of OPN in HSCs. (A-C) siRNA abrogation of Sox9 in activated rHSCs (A and B) and LX2 cells (C) standardized against scrambled siRNA control for mRNA (A) and protein (B and C). Example immunoblotting is shown as inset in (B) and (C). *p < 0.05; **P < 0.01; †*P* < 0.005; ‡*P* < 0.001, compared to control. (D) Alignment of the upstream *OPN* enhancer region with conserved SOX9-binding motif highlighted in black (human sequence shown is −3,886 to −3,842 base pairs relative to transcriptional start site). Conserved nucleotides indicated by asterisk (*). The core SOX-binding motif is CAAT with increased binding affinity for SOX9 conferred by additional flanking nucleotides.^49^ (E) ChIP assay for SOX9-binding element in conserved upstream *OPN* enhancer element in LX2 cells cultured in high serum and activated rHSCs. Negative control is immunoglobulin G (IgG), and positive control is input (diluted 10-fold).

To determine whether Sox9 regulates *Opn* expression, we abrogated Sox9 using siRNA in activated rHSCs. Reducing Sox9 by 70%-80% caused a commensurate 50%-70% decrease in *Opn* transcript and its encoded protein (Fig. 5A,B). Similar results were detected in the LX2 HSC line (Fig. 5C). *In silico* analysis of the *OPN* 5′ flanking region identified a conserved SOX9 binding motif ∼3 kilobase pairs upstream of the transcriptional start site (Fig. 5D). ChIP demonstrated that Sox9 was enriched at this site in both activated rHSCs and human LX2 cells (Fig. 5E; negative control data for *GAPDH* shown in Supporting Fig. 3). These data indicate that OPN is a novel downstream target of SOX9. Because others have implicated the Hh pathway in liver fibrosis^33^ and as a regulator of *OPN* expression,^14^, ^15^ and because in different circumstances SOX9 has been reported downstream of Hh signaling,^18^ we were curious to investigate whether SOX9 might be regulated by Hh in stellate cells as a means of determining OPN production.

### Hh Signaling Regulates SOX9 and Its Downstream Target, OPN

**Fig. 6:**
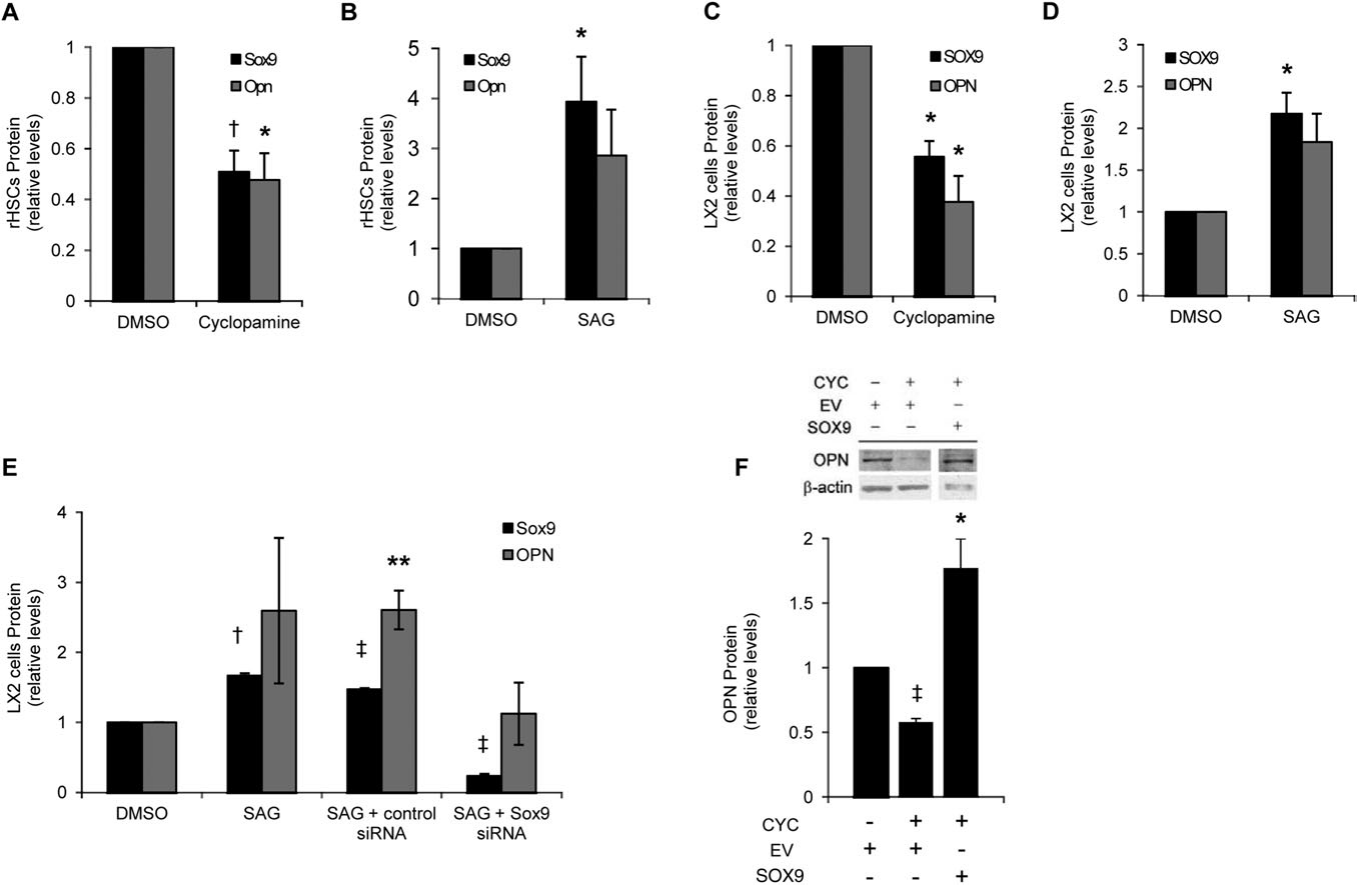
Hh regulates SOX9 and OPN expression in HSCs. (A-D) SOX9 and OPN protein levels quantified from immunoblotting of activated rHSCs and LX2 cells after 24-hour treatment with the Hh antagonist, CYC, or the Hh agonist, SAG. (E) Protein levels for SOX9 and OPN after treatment with SAG for 24 hours and knockdown of SOX9 (by 87%) using siRNA or scrambled control in LX2 cells. (F) Quantification of OPN protein after overexpression of SOX9 in LX2 cells in the presence or absence of CYC. Example immunoblotting image shown in inset. Change in expression is compared to vehicle treated cells (DMSO) for all experiments and, in the case of (F), EV control. Experiments standardized against β-actin. *P < 0.05; **P < 0.01; †*P* < 0.005; ‡*P* < 0.001, compared to control.

Serum-activated LX2 cells and rHSCs activated in culture for 10 days were incubated with the Hh antagonist, CYC, or agonist, SAG (Fig. 6A-D and Supporting Fig. 4A,B). Both SOX9 and OPN proteins were significantly decreased by 45%-60% in response to CYC and increased ∼2- to 3-fold after SAG treatment in both stellate cell models. These data demonstrate that both OPN and SOX9 are positively regulated by Hh signaling in stellate cells. To intimate a role for SOX9 as the mediator of Hh's effect on OPN production, we used siRNA in LX2 cells after SAG augmentation of Hh signaling (Fig. 6E and Supporting Fig. 4C). SAG induced increases in both SOX9 and OPN protein, compared to dimethyl sulfoxide (DMSO) control, which were unaffected by control siRNA. However, siRNA abrogation of SOX9 prevented the Hh agonist from increasing OPN levels above DMSO control values. To perform the converse experiment, transient transfection of an expression vector containing the human *SOX9* coding sequence was carried out to overexpress *SOX9* in LX2 cells (Supporting Fig. 4D). Overexpression of SOX9 rescued the inhibitory effect of CYC on OPN production (Fig. 6F). Collectively, these data implicate SOX9 as a positive regulator of OPN production downstream of Hh signaling in stellate cells.

### The Hh Mediator, GLI2, Regulates SOX9 Expression

**Fig. 7:**
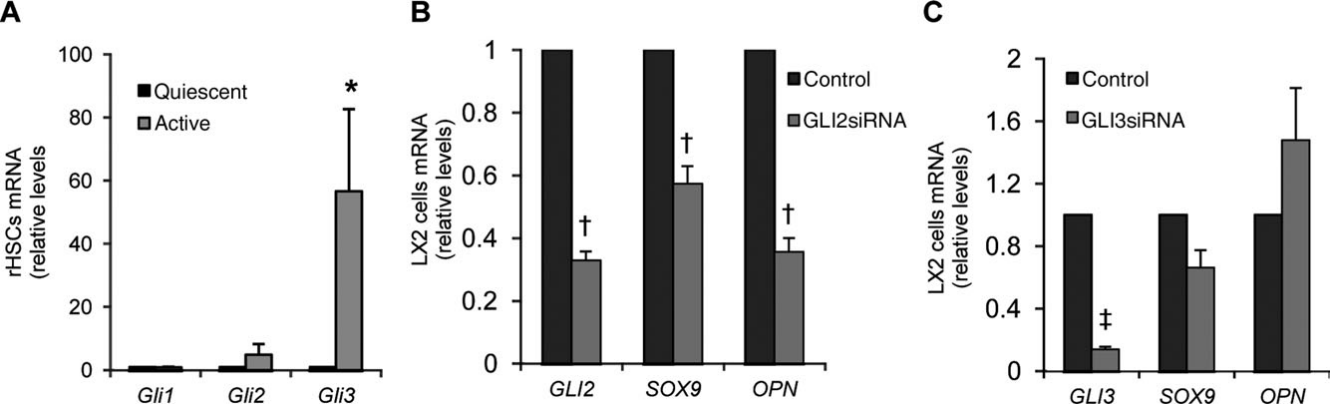
Gli2 mediates the expression of Sox9 and Opn in HSCs. (A) Expression of *Gli* factors in quiescent and activated rHSCs by qPCR. (B and C) siRNA for GLI2 (B, 67% knockdown) and GLI3 (C, 86% knockdown) or scrambled control in LX2 cells, followed by qPCR for GLI2 (B) or GLI3 (C), SOX9, and OPN. *P < 0.05; †*P* < 0.005; ‡*P* < 0.001, compared to scrambled siRNA treatment.

**Fig. 8:**
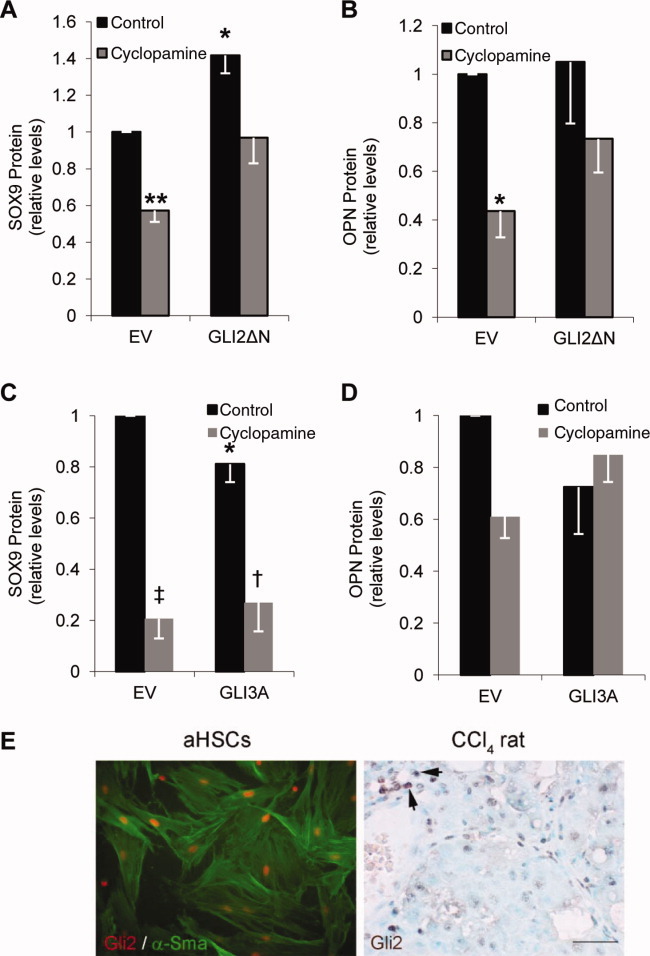
Gli2 overexpression rescues antagonistic effects of CYC on the expression of Sox9 and Opn in HSCs. (A-D) Quantification of SOX9 and OPN protein after overexpression of constitutively active GLI2 (GLI2ΔN; A and B) or active GLI3 (GLI3A; C and D) in LX2 cells in the presence or absence of CYC. (E) IF showing nuclear Gli2 (red) and cytoplasmic α-Sma (green) in activated rHSCs (aHSCs) and bright-field IHC showing nuclear Gli2 (brown staining) in CCl_4_-treated fibrotic rat liver. Arrows indicate Gli2 expression in a bile duct. *P < 0.05; **P < 0.01; †*P* < 0.005, ‡*P* < 0.001, compared to EV transfection. Size bar represents 20 μm.

The GLI family of transcription factors is known to mediate the effects of Hh signaling.^16^ To determine which GLI factor might be responsible for Hh's effect on SOX9 expression, we first investigated the expression of family members in quiescent and activated rHSCs. By qPCR, *Gli1* was poorly detected in quiescent HSCs and unaltered upon activation (Fig. 7A). In contrast, *Gli2* and *Gli3* mRNAs were increased ∼6- and ∼50-fold, respectively, in activated cells. Although, by this analysis, *GLI3* appears the more likely candidate for the regulation of *SOX9* in stellate cells, detection of mRNA is not indicative of protein levels, especially given the potential for both repressor or activator forms of GLI2 and GLI3. Several commercial and published antibodies were available to us^29^, ^34^, ^35^; however, we found them unhelpful in detecting or distinguishing the different forms by immunoblotting. Therefore, we investigated the potential contribution of GLI2 and GLI3 to *SOX9* and *OPN* expression by using siRNA in LX2 cells (Fig. 7B,C). Diminution of *GLI2* transcripts by ∼67% significantly reduced *SOX9* and *OPN* expression by ∼43% and ∼64%, respectively (Fig. 7B). In comparison, although achieving more robust reduction in *GLI3* expression (∼86%) with siRNA, *SOX9* expression was less affected and *OPN* was unaltered (Fig. 7C). Moreover, overexpression of constitutively active GLI2 (GLI2ΔN) was able to rescue, at least partially, the antagonistic effects of CYC on SOX9 and OPN production (Fig. 8A,B and Supporting Fig. 5A). In contrast, overexpressing the activator form of GLI3 (GLI3A) in the presence of CYC had little or no positive effect on SOX9 or OPN production (Fig. 8C,D and Supporting Fig. 5B). These data imply that GLI2 is the predominant regulator of *SOX9* expression in HSCs. In keeping with these results, nuclear immunoreactivity against Gli2 was detected in activated rHSCs *in vitro* and in regions of fibrosis after CCl_4_ treatment *in vivo* (Fig. 8E). Interestingly, Gli2 was also detected in the round nuclei of biliary epithelial cells similar to Sox9 and Opn (Fig. 8E, arrows). In contrast, despite detecting nuclear immunoreactivity for Gli3 in control brain tissue during human fetal development, such staining was not apparent in fibrotic rat tissue (data not shown).

## Discussion

OPN has been implicated as an important mediator, by which the inflammatory response ultimately leads to scarring and fibrosis in various organs,^6^-^10^, ^14^ with the potential that its presence in the circulation can be used as a biomarker of disease progression.^11^-^13^ Previously, we demonstrated a novel role for the transcription factor, SOX9, in models of liver fibrosis. Under the influence of transforming growth factor-beta (TGF-β) signaling, SOX9 became expressed in activated HSCs, where it was responsible for the production of the profibrotic collagen, COL1.^17^ In this study, we have demonstrated a more diverse role for SOX9 by regulating *OPN* expression. In the liver, SOX9 and OPN colocalized in the regions of fibrosis. The onset of OPN production during rHSC activation, its reduction in activated HSCs after Sox9 abrogation, and the binding of SOX9 to an upstream *OPN* enhancer element infers that the transcription factor is required for *OPN* expression during liver fibrosis.

SOX9 was responsive to Hh signaling in our models of liver fibrosis. Although Hh's precise role *in vivo* during liver fibrosis remains incompletely understood,^36^, ^37^ the signaling pathway is reactivated after injury in adult tissues^38^ and HSCs can produce and respond to Hh ligands.^30^, ^39^ Several lines of evidence place SOX9 downstream of Hh signaling. SOX9 is up-regulated by Hh ligands during chondrogenesis and, in neural stem cells, skin and intestine.^18^ The GLI family of zinc-finger transcription factors mediates Hh signaling in mammals.^16^ GLI1 is generally thought to be a transcriptional activator, whereas GLI2 and GLI3 have additional potential N-terminal repressor functions after proteolytic cleavage. There are several conserved GLI-binding motifs important for *SOX9* expression in its extended 5′ flanking region (up to 1.1 Mb).^18^, ^40^ Whereas Gli1 seems important for *Sox9* expression during chondrogenesis^41^ and for a SOX9-independent effect on *OPN* expression in malignant melanoma,^15^ the transcription factor was poorly detected in our models of liver fibrosis. In contrast, Gli2 increased Sox9 during mouse pancreatic β-cell dedifferentiation.^42^ Here, we demonstrate a role for GLI2 in regulating *SOX9* and *OPN* in models of liver fibrosis. This is in line with the detection of hepatic Gli2 by others.^33^ However, compared to a direct effect of GLI2 on *OPN*, our collective data indicate that GLI2 functions significantly through SOX9 in its regulation of OPN production. This mechanism may also extend to the up-regulation of *SOX9* by TGF-β, because GLI2 is induced by TGF-β in several cell types, including fibroblasts, keratinocytes, and cancer cells.^43^

In the healthy liver, both SOX9 and OPN localize to the bile ducts. SOX9 is required for normal biliary formation and function.^44^, ^45^ From our data, it seems likely that SOX9 would also be responsible for OPN production by healthy cholangiocytes. Furthermore, based on SOX9′s additional roles in regulating both COL1^17^ and collagen type 4 (COL4),^23^ there is the potential that SOX9 could be important in chronic cholestatic liver injury by regulating all these ECM components as part of the pathology of primary biliary cirrhosis (PBC) and primary sclerosing cholangitis. Interestingly, TGF-β and Hh signaling (including Gli2), both of which would up-regulate SOX9, have been implicated in the fibrotic response of PBC,^46^, ^47^ where COL1, COL4, and OPN are all increased.^48^

In summary, these data expand the role for SOX9 in regulating components of the ECM and begin to provide insight into its regulation by signaling pathways linked to fibrosis and related pathologies in the liver and other sites, such as the skin, kidney, lung, and major blood vessels.^18^ Finally, given the potential use of serum OPN as a biomarker for the severity of liver damage in patients with HBV or HCV,^11^, ^12^ it is possible that additional downstream ECM targets of SOX9 action may be useful in helping to stage and predict the severity of liver fibrosis.

## Abbreviations

α-SMA: alpha-smooth muscle actin
cDNA: complementary DNA
ChIP: chromatin immunoprecipitation
Col1: collagen type 1
Col4: collagen type 4
CYC: cyclopamine
DMSO: dimethyl sulfoxide
ECM: extracellular matrix
EV: empty vector
GLI: glioblastoma
HCV: hepatitis C virus
HSC: hepatic stellate cell
hHSCs: human HSCs
Hh: Hedgehog
ICC: immunocytochemistry
IF: immunofluorescence
IHC: immunohistochemistry
mRNA: messenger RNA
OPN: osteopontin
PBC: primary biliary cirrhosis
qPCR: quantitative polymerase chain reaction
rHSCs: rat hepatic stellate cells
SAG: smoothened agonist
siRNA: short interfering RNA
SOX9: sex-determining region Y-box 9
TGF-β: transforming growth factor-beta

## Supplementary material

Supporting Information Figure 1. Immunohistochemistry of SOX9 and OPN in normal mouse liver. Consecutive 5 μm sections of normal liver in mouse stained for SOX9 and OPN (brown), counterstained with toludine blue. Detection only in the round nuclei (SOX9) and cytoplasm (OPN) of biliary epithelial cells. Size bar represents 50 μm

Supporting Information Figure 2. Sox9 and Opn detection in activated HSCs. A, Example immunoblot showing increased Sox9 and Opn in activated (A) compared to quiescent (Q) rat HSCs (rHSCs). B, Quantification of Sox9 and Opn in quiescent and activated rHSCs detected by immunoblotting. Immunoblotting quantification was normalized to β-actin. **, p<0.01, -, p<0.005.

Supporting Information Figure 3. PCR for an intergenic region of GAPDH (i.e. no Sox9 binding motif) as a negative control for the ChIP assay shown in Figure 4. Positive control is input (diluted 10-fold). No PCR product is seen following immunoprecipitation using anti-Sox9 (or negative control IgG) antibodies.

Supporting Information Figure 4. A and B, Example immunoblots showing decrease and increase in OPN and SOX9 following cyclopamine or SAG treatment respectively (relating to experiments in Fig. 6B and D). C, Example immunoblot for Fig. 6E). D, Control data for Fig. 6F to demonstrate that transient transfection of the SOX9 cDNA expression vector produces increased levels of SOX9 in the presence of cyclopamine (CYC) in LX2 cells. Quantification of SOX9 protein was normalized to β-actin. -, p<0.005 compared to empty vector (EV) / DMSO treated control.

Supporting Information Figure 5. A and B, example immunoblots and verification of myc-tagged GLI2ΔN and GLI3A expression in cells following transfection. EV, empty vector control.
