# Supplementary figures and images for: Osteopontin Is a Novel Downstream Target of SOX9 With Diagnostic Implications for Progression of Liver Fibrosis in Humans
Source: Hepatology. 2012 Sep;56(3):1108–16. doi: 10.1002/hep.25758 (PMC3638324; doi:10.1002/hep.25758)

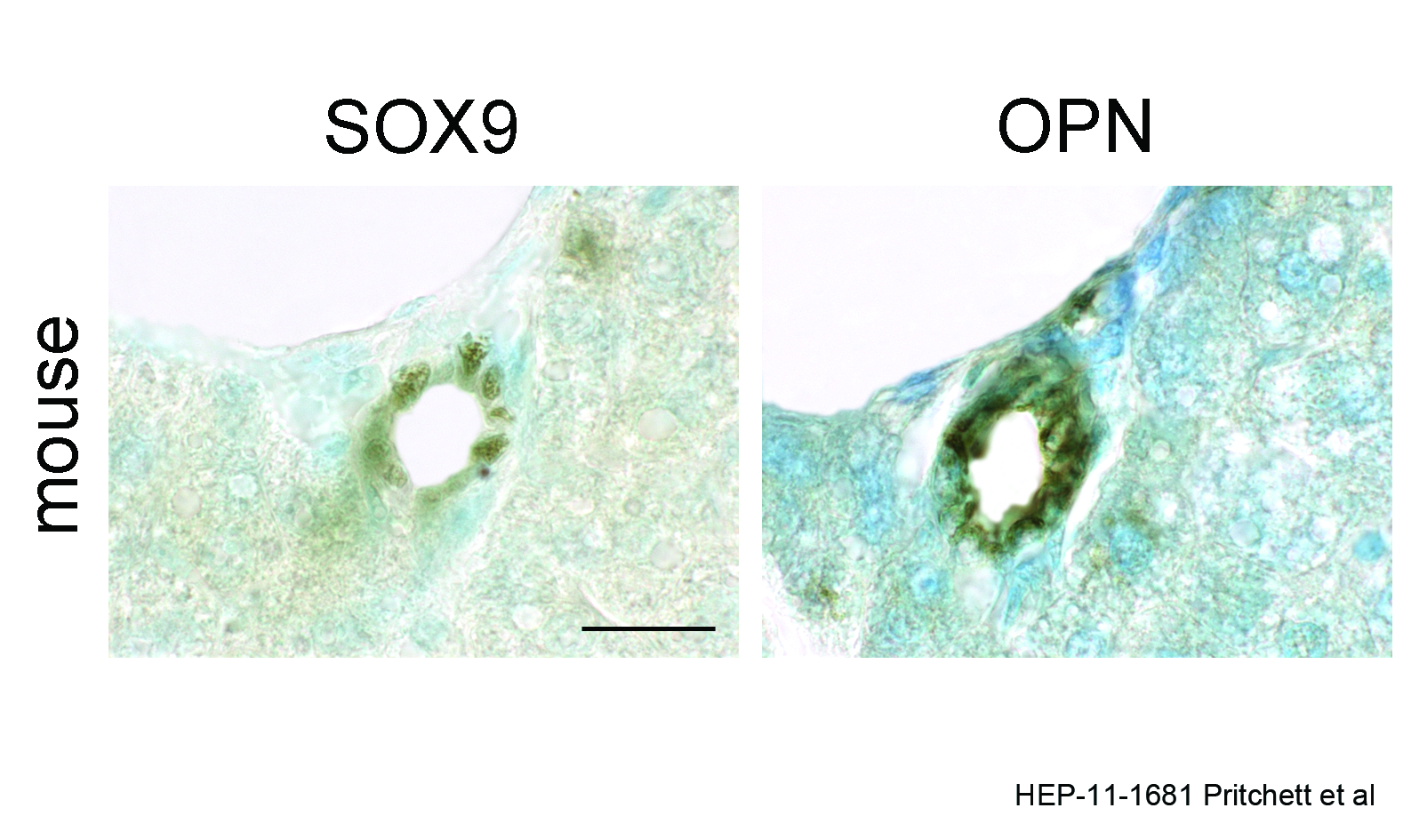

Supplement: Supplementary file 1 [file hep0056-1108-SD1.tif]

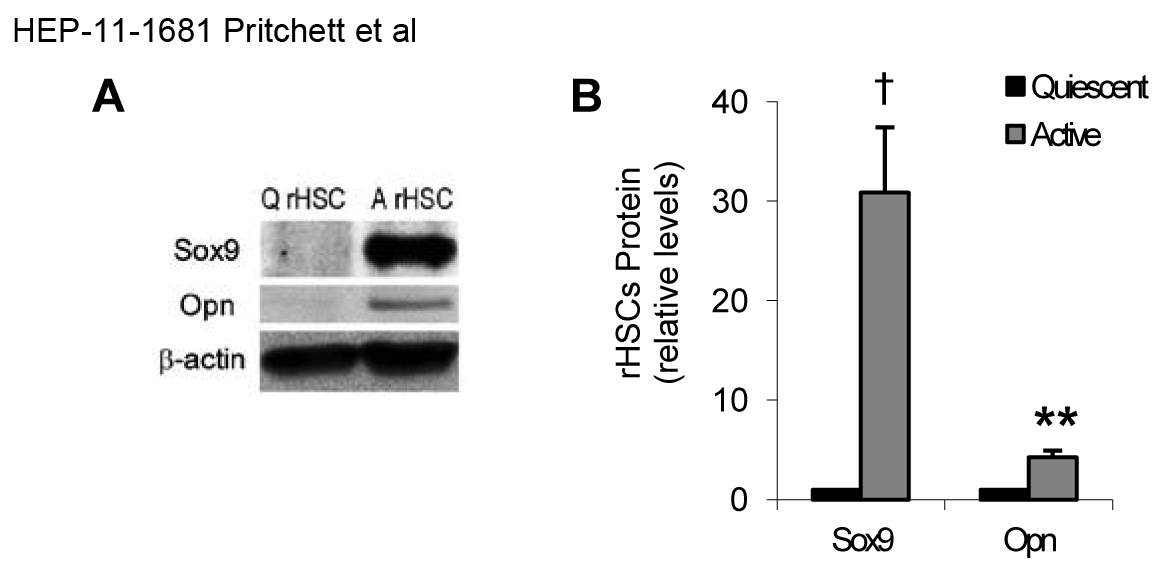

Supplement: Supplementary file 2 [file hep0056-1108-SD2.tif]

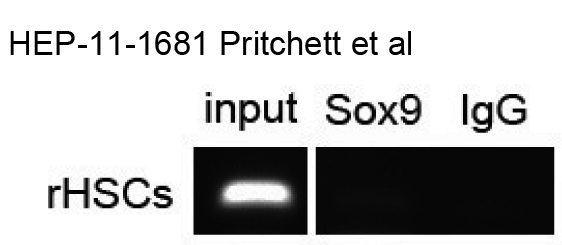

Supplement: Supplementary file 3 [file hep0056-1108-SD3.tif]

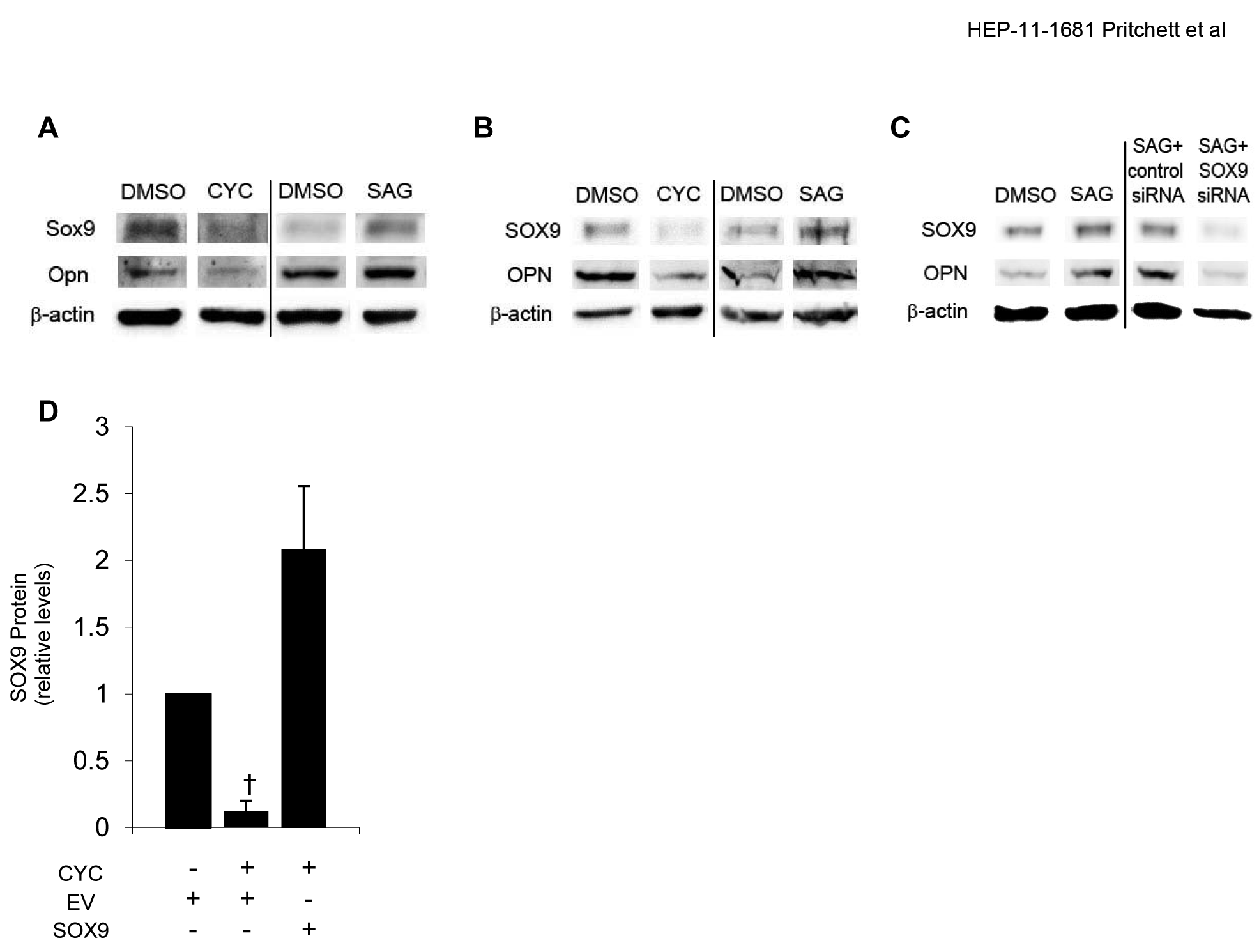

Supplement: Supplementary file 4 [file hep0056-1108-SD4.tif]

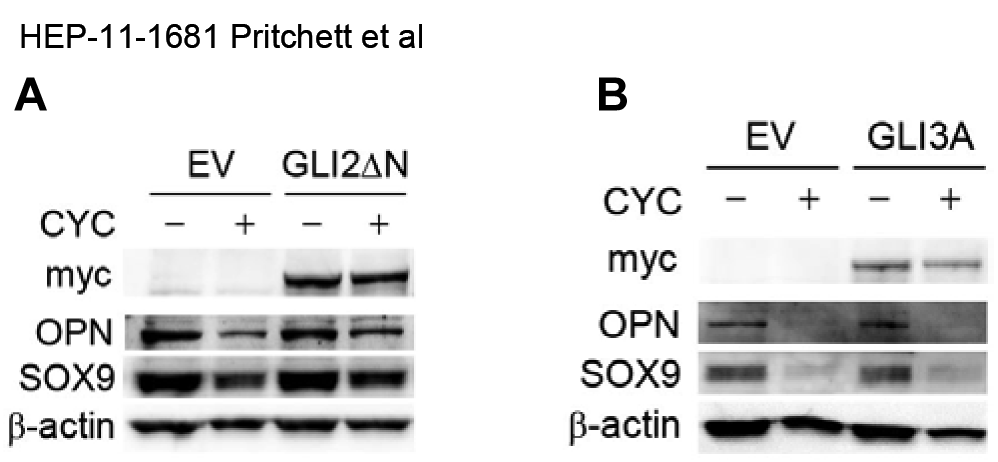

Supplement: Supplementary file 5 [file hep0056-1108-SD5.tif]
