# Supplementary material for: Osteopontin Is a Novel Downstream Target of SOX9 With Diagnostic Implications for Progression of Liver Fibrosis in Humans
Source: Hepatology. 2012 Sep;56(3):1108–16. doi: 10.1002/hep.25758 (PMC3638324; doi:10.1002/hep.25758)
Supplement: Supplementary file 6 [file hep0056-1108-SD6.doc]

## Supplementary Methods

***Isolation and culture of human HSCs.*** Human liver tissue was obtained from patients undergoing partial liver resection for metastatic cancer with full ethical approval and following written informed consent from each patient. Briefly, liver resections were perfused *ex-vivo* with Hanks HEPES buffer containing EGTA (GIBCO-BRL, Life Technologies, UK), followed by perfusion with Hanks HEPES buffer without EGTA. Tissue was then digested for 20 min with Hanks HEPES buffer containing calcium, collagenase (0.13U/ml NB4G grade collagenase, SERVA Electrophoresis GmbH, Heidelberg, Germany) and trypsin inhibitor (Sigma, UK). The digested tissue was minced with blunt forceps and scissors, agitated gently in Hanks HEPES buffer without EGTA and filtered through 250µm and 100µm nylon membranes. The resultant cell suspension was spun down at 700 rpm for 2 minutes and washed twice in Williams’ medium E (GIBCO-BRL, Life Technologies, UK) containing 10% heat inactivated foetal calf serum (Sigma, UK), and 2mM L-glutamine (Sigma, UK). Supernatants from centrifugal spins of the hepatocyte isolation process were pooled and retained for HSC isolation as follows: supernatants were centrifuged 3 times at 50 g for 5 minutes until no pellet was formed, then centrifuged at 4000g for 20 minutes to form a pellet containing HSC. The supernatant was discarded and the pellet resuspended in 20ml of Dulbecco’s Modified Eagles Medium (DMEM) (GIBCO-BRL, UK) containing 10% FCS and seeded in a 75cm^2^ tissue culture flask. Medium was changed 24 hours after seeding and every 48 hours thereafter. HSC acquired the fully activated phenotype and became confluent after 7-10 days. HSC isolated using this method have been characterised by morphology, the presence of intracellular lipid, and staining for HSC specific markers α-smooth muscle actin (α-SMA) and glial fibrillary acidic protein (GFAP) by immunohistochemistry. Confluent HSC were detached by trypsinisation and seeded at a density of 3 x 10^5^ cells per well on 6 well plates.

***Chromatin immunoprecipitation (ChIP) assays.*** LX2 cells or rat HSCs were fixed in 1% formaldehyde in DMEM for 10mins, and washed in PBS. After 10mins incubation in 0.125M glycine, cells were scraped into PBS with 1.25mM PMSF. Following centrifugation cell pellets were resuspended and lysed in cell lysis buffer (10mM HEPES pH6.5, 0.5mM EGTA, 10mM EDTA, 0.25% Triton X-100) for 5mins at 4°C. After further centrifugation, pelleted nuclei were washed in ice cold nuclei wash buffer (10mM HEPES pH6.5, 0.5mM EGTA, 10mM EDTA, 200mM NaCl), centrifuged and lysed in ice cold nuclei lysis buffer (50mM Tris-HCl pH 8.1, 10mM EDTA, 1% SDS). Chromatin was sheared by sonication to generate 200bp – 1000bp fragments. Sheared chromatin was incubated overnight at 4°C with 3μg antibody (see Supplementary table 3) and magnetic protein G coated beads (precleared with sheared salmon sperm and BSA). Antibody-bead complexes were washed 3 times in each of the following buffers: Buffer 1 (20mM Tris-HCl pH8.1, 2mM EDTA, 50mM EDTA, 0.1% SDS, 1% Triton X-100), Buffer 2 (10mM Tris-HCl pH8.1, 1mM EDTA, 250mM LiCl, 1% NP-40, 1% Deoxycholate), and TE Buffer (10mM Tris-HCl pH8.1, 1mM EDTA). Protein-DNA complexes were eluted by shaking in elution buffer (1% SDS, 100mM NaHCO_3_) for 45mins at room temperature. Protein-DNA crosslinks were reversed by incubation for 2.5 hours at 65°C following the addition of 200mM NaCl. DNA was purified using MinElute columns (QIAGEN) and analysed by PCR (see Supplementary table 4).
