# Supplementary material for: Osteopontin Is a Novel Downstream Target of SOX9 With Diagnostic Implications for Progression of Liver Fibrosis in Humans
Source: Hepatology. 2012 Sep;56(3):1108–16. doi: 10.1002/hep.25758 (PMC3638324; doi:10.1002/hep.25758)
Supplement: Supplementary file 7 [file hep0056-1108-SD7.doc]

### Supplementary Table 1. siRNA.

| Species | Target | Sequence | Supplier | Catalogue Number |
| --- | --- | --- | --- | --- |
| Human | *SOX9* | ATG GGA GTA AAC AAT AGT CTA | Qiagen | SI00007595 |
| Human | *SOX9* | TCG TGT GAT CAG TGT GCT AAA | Qiagen | SI00007609 |
| Human | *GLI2* | TAG GGA GCA TTT GGG TTT GAA | Qiagen | SI00074844 |
| Human | *GLI2* | CTC GCT AGT GGC CTA CAT CAA | Qiagen | SI03091445 |
| Human | *GLI3* | CAC CAT ATA AAT AAC GAC CAT | Qiagen | SI00003570 |
| Human | *GLI3* | CCG CCT TAT CTA GTA GCC CTA | Qiagen | SI00003584 |
| Rat | *Sox9* | GAG AGA GAC TTT AAG ACA TTA | Qiagen | SI02952663 |
| Rat | *Sox9* | CCC ACC AGC GTC AGT GAG GAA | Qiagen | SI02952677 |

### Supplementary Table 2. Vectors.

| Vector | Source |
| --- | --- |
| pcDNA3.1zeo+ | Promega |
| pcDNA3.1zeo+ SOX9FL | Subcloned from gifted plasmid Vincent Harley |
| pCS2-MT-Myc | Gifted plasmid from Ruiz i Altaba (29, 30) |
| pCS2-MT GLI2∆N-Myc | Addgene (29) |
| pCS2-MT GLI3A-Myc | Gifted plasmid from Ruiz i Altaba (30) |

### Supplementary Table 3. Antibodies.

| Antibody | Raised In | Company | Dilution | Application* |
| --- | --- | --- | --- | --- |
| SOX9 | Rabbit | Millipore | 1:5000  1:800 | Western  IHC / ICC |
| SOX9 | Rabbit | Santa Cruz | - | ChIP |
| OPN | Goat | Abcam | 1:750 | Western |
| OPN | Mouse | Leica/Novocastra | 1:50 | ICC |
| OPN | Mouse | R&D Systems (AF808) | 1:80 | IHC (Pepsin Digest) |
| OPN | Goat | R&D Systems (AF1433) | 1:80 | IHC (Pepsin Digest) |
| α-SMA | Mouse | Leica/Novocastra | 1:100  1:50 | IHC  ICC |
| Gli2 | Rabbit | Genway | 1:400 | ICC |
| β-actin | Mouse | Sigma | 1:100 000 | Western |
| Col I | Rabbit | Gift from Dr Larry Fisher, NIH,  Bethesda, MD (1) | 1:2000 | Western |
| Myc | Mouse | Sigma | 1:150 | Western |

*For IHC and ICC antigen retrieval was performed by boiling in 6mM Sodium Citrate for 10mins (IHC) or 5mins (ICC), unless stated otherwise.

1. Fisher LW, Stubbs JT, 3rd, Young MF. Antisera and cDNA probes to human and certain animal model bone matrix noncollagenous proteins. Acta Orthop Scand Suppl 1995;266:61-65.

### Supplementary Table 4. PCR primers.

| Gene | Species | Forward | Reverse | Application | Product Length |
| --- | --- | --- | --- | --- | --- |
| *Sox9* | Rat | gcaagtagccctggtttcgttctc | gggtggccagtgctcagttgc | qPCR | 99 |
| *Opn* | Rat | gagtttggcagctcagagga | tctgcttctgagatgggtca | qPCR | 91 |
| *Opn* | Rat | agtctatggaaaggaggcagtatt | ttcagaggtgggttttgttgtt | ChIP | 149 |
| *Gli1* | Rat | ctggaggtctgcgtggtag | catggtgtctcagcgaagg | qPCR | 60 |
| *Gli2* | Rat | actgaggctgagggcctac | gagatcagccagttgctcct | qPCR | 66 |
| *Gli3* | Rat | accaaaacagaacacattccaa | ggggtctgtgtaacgcttg | qPCR, | 77 |
| *GusB* | Rat | ctctggtggccttacctgat | aggtgttgtcatcgtcacctc | qPCR, | 72 |
| *SOX9* | Human | gtacccgcacttgcacaac | tcgctctcgttcagaagtctc | qPCR | 72 |
| *OPN* | Human | gcttggttgtcagcagca | tgcaattctcatggtagtgagttt | qPCR | 127 |
| *OPN* | Human | gcacttactccataacagttctatgtg | cattgctcaatagcttagacaacaa | ChIP | 135 |
| *GUSB* | Human | ctcatttggaattttgccgatt | ccgagtgaagatccccttttt | qPCR | 81 |
| *Gapdh* | Rat | tagctggcctggtgataac | ggggccccaaggaggtagg | ChIP | 113 |

### 
